# Differences in Gender Norms Between Countries: Are They Valid? The Issue of Measurement Invariance

**DOI:** 10.1007/s10680-014-9329-6

**Published:** 2014-09-24

**Authors:** Dorota Weziak-Bialowolska

**Affiliations:** European Commission—Joint Research Centre, Deputy Directorate-General, Econometrics and Applied Statistics Unit, Via E. Fermi 2749, TP 361, 21027 Ispra, VA Italy

**Keywords:** Gender norms, Gender attitude, Gender equality, Measurement invariance, Multi-group confirmatory factor analysis with alignment

## Abstract

The values and attitudes towards gender roles are often investigated and compared from a cross-country perspective without the proper statistical treatment of the measurement invariance (MI) assessment. This implies that the conclusions based on composite scales of gender norms, gender role attitudes or gender egalitarianism, to name only a few, may be questionable. In this study, we address this lack by investigating the cross-country MI properties of the Gender Equality Scale (GES) based on World Value Survey data. We use multi-group confirmatory factor analysis with and without alignment to determine the configural, weak, strong and strict MI. The results show that the concept of gender equality is not comparable across all countries involved in the survey. In particular, it seems to differ between Western Europe and Central and Eastern Europe. We claim that only selected Central and Eastern European countries exhibit a configural MI but fail to show full weak MI and definitely fail to show full strong and full strict MI. However, under the aligned measurement framework, we succeeded in showing that for these countries, comparisons of the country rankings with respect to the GES are valid provided that a correction for non-invariance of certain factor loadings and/or intercepts is applied. Our study shows that the most egalitarian gender role attitudes measured by the GES are observed in the Czech Republic, Hungary, Lithuania and Croatia. They are significantly higher than the gender equality attitudes recorded in the lowest scoring countries Poland, Slovakia, Albania and Romania.

## Introduction

The model of gender norms lacks an unequivocal conceptualisation. Questions regarding this concept are addressed differently in various surveys, such as the Population Policy Acceptance Study (PPAS), the World Value Survey (WVS) and the International Social Survey Programme (ISSP). Thus, the concept is operationalised differently, and its measurement scale is usually a product of either exploratory multivariate statistical techniques (principal component analysis, classical—in the literature called exploratory—factor analysis) or, much less frequently, classification methods.

In addition to the purely conceptual problems associated with the measurement of gender norms reported by various authors, including Davis and Greenstein (2009), Goldscheider et al. (2010) and Westoff and Higgins (2009), the increasing complexity of studies that aimed to investigate the effects of gender norms on different socio-economic phenomena across countries raises the question of their concept equivalence and concept measurement invariance (MI) properties (Byrne et al. 1989; Byrne and van de Vijver 2010; Davidov et al. 2008b; Gregorich 2006; Meredith 1993). This problem is not trivial because measurement non-invariance constitutes one of the most serious threats to cross-cultural research (Davidov et al. 2012) by introducing bias into the assessment of both the strength of relationships (correlations) and the level comparisons of the latent phenomenon of interest (country rankings). In a study conducted using questionnaire data from different countries, MI ensures that in all measurements and in all of the subpopulations, both the concepts measured have the same meaning, and the same measurement unit and reference point of the measurement scale are used. Thus, MI enables researchers to make meaningful comparisons of the different constructs of interest.

This type of research has been conducted in various fields, including social sciences (Bialowolski and Weziak-Bialowolska 2013; Davidov et al. 2008b; Raijman et al. 2008; Weziak-Bialowolska 2010), educational research (Byrne et al. 1989; Raykov et al. 2012), psychology (Coertjens et al. 2012; Reise et al. 1993), organisational research (Bialowolski and Weziak-Bialowolska 2014; Vandenberg and Lance 2000) and medical care (Meredith and Teresi 2006). However, to the best of our knowledge, no such test has been performed on the analysis of the gender norm scales. The only exception we found was the study conducted by Aboim (2010), who performed tests of construct equivalence (i.e. of configural invariance). This finding implies that the conclusions based on composites, composite indexes or composite scales of gender norms, gender ideology, gender roles attitudes, gender-related attitudes or gender egalitarianism, to name only a few, may be different.

Thus, the aim of this paper was to address this lack by investigating the MI (measurement equivalence) properties of the Gender Equality Scale (GES) (Inglehart and Norris 2003) based on the WVS data. We tested these properties based on country identifier. However, we highlight that such an analysis might also be conducted with respect to each classification variable of interest (e.g. gender, age group, marital status and measurement occasions) and with respect to each latent and complex concept. Therefore, although the paper focuses on gender equality concept, it can be of interest also for researches from other fields.

In the following sections, we present the analysis background and the data sources used. Because we found no research study on gender role attitudes that implemented any of the MI concepts or methodologies, we present these concepts concisely in the section titled *Testing for MI*. The modelling strategy and results, with special emphasis on the Central and Eastern European (CEE) countries, are discussed after that and the main conclusions are provided in the final section.

## Background

Studies testing the influence of the gender norm concept on different phenomena are numerous. In the field of demography, the effects of gender norms on work-family conflict (Marler and Moen 2005; Weer et al. 2006), the division of housework (Chesters 2010), fertility (McDonald 2013; Muszyńska 2007; Philipov 2008; Westoff and Higgins 2009) and women’s employment (Cunningham 2008; Marler and Moen 2005; Motiejunaite and Kravchenko 2008; Motiejunaite 2008) are of special interest. In these studies, two approaches can be distinguished for the comparison of associations between gender norms and other phenomena. In the first approach, the authors focus on making comparisons of the relationship between gender norms and other phenomena across measurement occasions (Chesters 2010; Cunningham 2008; Kaufman 2000; Motiejunaite and Kravchenko 2008; Motiejunaite 2008), countries (Motiejunaite and Kravchenko 2008; Muszyńska 2007; Philipov 2008) or subpopulations (Kroska and Elman 2009; Lucier-Greer and Adler-Baeder 2011). In contrast, the second approach involves the establishment of country classifications with respect to gender role attitudinal regimes (Bejarano et al. 2012; Luck and Hofacker 2003; Motiejunaite 2008; Treas and Widmer 2013), which are further contrasted with empirical findings on the interrelationship between fertility and women’s employment.

Previous research on the relationship between gender norms and fertility and/or labour market outcomes across countries or subpopulations have generally shown that societies are more egalitarian with respect to gender norms nowadays than they used to be 20 years ago (Chesters 2010; Lucier-Greer and Adler-Baeder 2011) and that more egalitarian gender norms are positively linked with women’s labour market outcomes in terms of employment (Cunningham 2008; Kroska and Elman 2009; McDonald 2013) and with men’s involvement in housework (Chesters 2010). In addition, egalitarian gender attitude evolves in the life course strengthening (1) in subsequent male and female birth cohorts (Chesters 2010), (2) in individuals divorced or remaining single compared to their own attitudes in first marital relationship and (3) in individuals between their first and following marital relationship (Lucier-Greer and Adler-Baeder 2011).

However, there are also inconsistent results presented in the literature. Puur et al. (2008) established a positive link between men’s fertility intentions and the egalitarian gender role attitudes regarding responsibilities for domestic tasks and childrearing, and these results were questioned by Westoff and Higgins (2009). Philipov (2008) reported mixed results regarding gender egalitarian attitudes and fertility. These researchers found that the effect of egalitarian gender ideology on fertility appears to be positive when referring to gender equality at home, such as the division of housework and care responsibilities, but negative when referring to gender equality in the public sphere, such as employment and political life (Goldscheider et al. 2010; Westoff and Higgins 2009). Nevertheless, this discussion led to the conclusion that there is a more systemic problem: a lack of consensus not only on the measurement of gender norms (Davis and Greenstein 2009; Westoff and Higgins 2009), but also on the understanding of the concept (i.e. gender equality and gender equity and their differential correspondence with different outcomes, such as fertility, as recently recalled by McDonald (2013)), including, as highlighted herein, the concept of equivalence in comparative studies.

Regarding the approach comprising country classifications, Muszyńska (2007) proposed the grouping of the European countries into six groups based on the work motivation of the women living in those countries (for money or for higher-order needs). Puur et al. (2008) classified seven European countries according to the perceptions of male social roles into countries with egalitarian, intermediate and traditional attitudes. Westoff and Higgins (2009) followed the same classification based on the GES (Inglehart and Norris 2003). Philipov (2008) presented a hierarchy of ten European countries with respect to the perception of the traditional gender roles and support for women’s employment, and Inglehart and Norris (2003) performed a similar study with respect to the GES.

The data sources used in the aforementioned studies were the Marital Instability over the Life Course database—Lucier-Greer and Adler-Baeder (2011), Social Structure of Australian Project 1986 and 1993—Chesters (2010), PPAS—Philipov (2008); Puur et al. (2008), WVS—Inglehart and Norris (2003); Westoff and Higgins (2009) and ISSP—Luck and Hofacker (2003); Motiejunaite and Kravchenko (2008); Muszyńska (2007). Of these sources, the results of Chesters (2010); Inglehart and Norris (2003); Lucier-Greer and Adler-Baeder (2011); Luck and Hofacker (2003); Motiejunaite and Kravchenko (2008); Muszyńska (2007); Philipov (2008); Puur et al. (2008); Westoff and Higgins (2009) were obtained using composite indices (composite scales), all of which were based on four- or five-point Likert statements that were summed (Inglehart and Norris 2003; Motiejunaite and Kravchenko 2008; Puur et al. 2008), averaged (Chesters 2010; Lucier-Greer and Adler-Baeder 2011; Philipov 2008) and/or used to calculate the principal component scores (Muszyńska 2007; Philipov 2008) or factor scores (Luck and Hofacker 2003). Only Inglehart and Norris (2003), Luck and Hofacker (2003), Motiejunaite and Kravchenko (2008) and Philipov (2008) verified the dimensionality of their indicators. Inglehart and Norris (2003), Luck and Hofacker (2003) and Philipov (2008) conducted it through principal component analysis, but these verifications were performed for the pooled dataset. Motiejunaite and Kravchenko (2008) used classical (exploratory) factor analysis and although conducted it for two analysed countries separately, entirely ignored the issue of obtaining solutions with different numbers of factors (see footnote 8 in Motiejunaite and Kravchenko 2008), which strongly implies a lack of concept comparability, i.e. a lack of configural invariance. The consistency of the indicators was verified by Chesters (2010); Inglehart and Norris (2003); Luck and Hofacker (2003); Motiejunaite and Kravchenko (2008); Westoff and Higgins (2009) through the application of Cronbach’s alpha, which may also be questioned (see Bentler 2009; Sijtsma 2009).

As mentioned above, the described indices have been used (1) in a regression framework (Inglehart and Norris 2003; Luck and Hofacker 2003; Philipov 2008; Westoff and Higgins 2009) to compare gender attitudes among different groups of people (Lucier-Greer and Adler-Baeder 2011; Philipov 2008) or countries (Muszyńska 2007; Philipov 2008) and (2) to classify different countries (Muszyńska 2007; Puur et al. 2008; Westoff and Higgins 2009). In all of these approaches, it is necessary to verify the measurement equivalence across the investigated groups (Byrne et al. 1989; Byrne and van de Vijver 2010; Byrne 2008; Meredith 1993; Meredith and Teresi 2006; Steinmetz et al. 2007; Vandenberg and Lance 2000; Wu et al. 2007). Unfortunately, none of these studies include this analysis which implies that, regardless of the aim, the researchers made the salient assumption that each composite gender norm scale exhibits measurement equivalence with regard to the country, measurement occasion and/or subpopulation, which may not be true. In some analysed countries, measurement occasions and/or subpopulations, it is likely that (1) the gender norm concept is understood differently (i.e. the perception of the content of the questions measuring the gender norm concept varies as well as meaning, understanding and/or relevance of the measured construct) and (2) its scale has different measurement units and (3) different reference points. This further implies that the same answer to a particular question is calibrated to different factor scores depending on the country, measurement occasion and/or subpopulation to which the respondent belongs to.

The reasons for non-invariance may be numerous. The most often quoted ones are Byrne and van de Vijver (2010); Rutkowski and Svetina (2013) of a technical nature: differential interpretation of Likert scale anchors, differential response style, differential familiarity with item scale format and translation errors or related to cultural and institutional bias: differential extent to which respondents from a particular country have inculcated its social values and mores.

## Testing for Measurement Invariance

To make valid comparisons of composite scale scores across countries, measurement occasions or sub-populations and to investigate the correlation between the composite scale and other variables of interest, it is necessary to establish the MI, also called the measurement equivalence, of a composite scale. It must be noted, however, that from a technical point of view, it is possible to calculate the mean factor score corresponding to the average level of the latent variable in a particular group or country using principal component analysis, classical (exploratory) factor analysis or confirmatory factor analysis without ascertaining measurement equivalence. Only after the MI is established a researcher can be confident that the scale scores from different countries, measurement occasions or subpopulations (henceforth called groups for simplicity) measure the same construct (ensuring the comparability of its meaning and understanding) using the same measurement unit (ensuring the same rating reference frame across groups).

Davidov (2008) reported that there are three major MI testing techniques: the differential item functioning approach (Jansen 2011), item response theory models (de Jong et al. 2007) and the factor analysis framework (Byrne 2008; Davidov et al. 2008a; Gregorich 2006; Wu et al. 2007). The most frequently used technique is multi-group confirmatory factor analysis (MG-CFA), and this technique was applied in our analysis.

Meredith (1993) distinguished four levels of MI: (1) configural invariance, (2) weak invariance, (3) strong invariance and (4) strict invariance. To these four types, Gregorich (2006) added another type that should precede them: dimensional invariance. In the factor analysis framework, dimensional invariance relates to the verification of the number of factors in each group through the exploratory factor analysis. Configural invariance refers to the verification of whether the same factor model (in terms of the number of both the factors and the observable variables associated with the factors) is well fitted across the groups compared (Horn and McArdle 1992). A weak MI requires the verification of whether the factor loadings are invariant across groups, and a strong MI is related to the assessment of whether a model has not only factor loadings but also factor intercepts that are identical across groups. A strict MI requires that a model with equal cross-group error variances, factor loadings and factor intercepts is well fitted.

Considering its typology, the process of establishing the MI is clearly hierarchical. This process most often starts with the establishment of a well-fitting baseline model for each group separately and then proceeds to the testing of subsequent types of MI to establish the following:

i. configural invariance: it ensures that common factors are associated with the same items across groups, which implies that the concept has the same cross-group meaning but is not sufficient for meaningful statistical comparisons;
ii. weak MI: it ensures that a one-unit difference in the question scores is calibrated to the same one-unit difference in the factor scores in all of the analysed groups. This weak MI further implies that any changes in the factor scores or scale scores have the same meaning across groups, and this effect is due to the fact that in all of the groups, the same measurement unit of a factor scale is guaranteed. Therefore, a researcher is entitled to compare across groups, the relationships between factor scores/scale scores and other observable variables. For example, only after establishing the existence of a weak MI, can the statement “egalitarian gender norms are positively linked with men’s involvement in housework, but this relationships is stronger in Scandinavian countries than in Southern European countries” be supported;
iii. strong MI: it implies that, in addition to the same one-unit difference in the question and factor scores in all of the analysed groups, the same answers (e.g. “I agree”) to a given question reported by respondents from different groups are calibrated to the same factor scores. Thus, if a researcher wants to conduct valid cross-group comparisons of the scale scores (e.g. country rankings based on mean scale scores), a strong MI is required. For example, only after establishing a strong MI, can the statements “egalitarian gender attitude evolves in the life course and are stronger in subsequent birth cohorts”, “egalitarian gender attitude is stronger in individuals divorced or remaining single compared to the attitude they had in their first marital relationship” and “egalitarian gender norms are stronger in Scandinavian countries than in Southern European countries” be supported;
iv. strict MI means that, in addition to that stated in (iii), the reliabilities of the scales that are indirectly reflected by error variances are comparable across groups. However, it must be noted that there is no consensus on whether a strict MI is necessary to perform valid cross-group comparisons of the mean scale scores. Lubke and Dolon (2003), Meredith (1993), Wu (2007) state that a strict MI is required, whereas Byrne and van de Vijver (2010), Davidov et al. (2008a), Davidov (2008) discuss that meaningful information can be obtained by assuming only a strong MI.

All types of invariance can be verified either fully or partially (Byrne et al. 1989; Byrne 2008; de Jong et al. 2007; Gregorich 2006; Millsap and Kwok 2004; Steenkamp and Baumgartner 1998). In the full version of MI, the equality constraints concern all of the manifest variables, whereas some of these can be relaxed in the partial version. Therefore, only a subset of the manifest variables that satisfy the strong or strict MI criteria is used to estimate the group differences (Gregorich 2006).

**Fig. 1:**
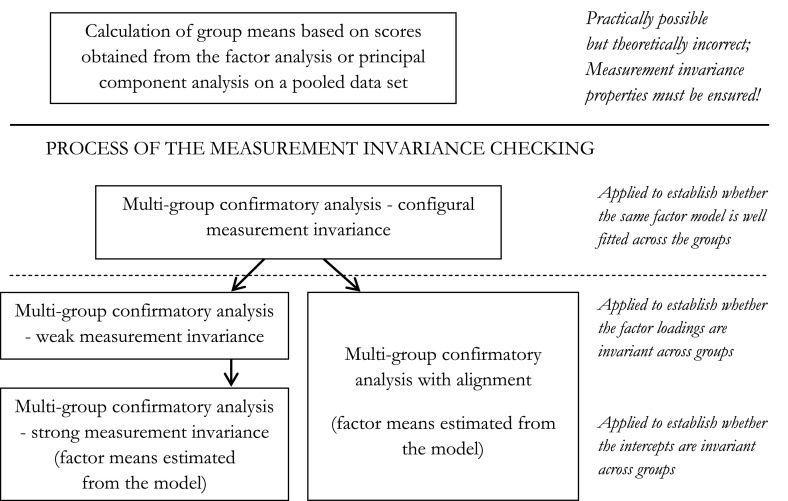
Steps in analysis aimed at comparing of factor means

When the MI is not satisfied, subgroups of countries that are measurement non-invariant may be sought (Davidov et al. 2008b; Welkenhuysen-Gijbels et al. 2003). However, in the large-scale cross-cultural studies in which the measurement instruments often do not demonstrate adequate measurement equivalence properties due to a large number of countries subject to assessment (examples can be found in Byrne and van de Vijver (2010); Davidov et al. (2008b); Rutkowski and Svetina (2013)), establishing which model parameters to relax is too cumbersome due to many possible violations of invariance and many possible modifications. These possible modifications, in turn, are likely to lead to the wrong—far from the true—model (Asparouhov and Muthén 2014). Therefore, Asparouhov and Muthén (2014) have recently proposed a new approach, namely the multi-group factor analysis with alignment, which accommodates the classical MG-CFA to the specificity of the large-scale (with a large number of compared groups, e.g. countries) international surveys. In this approach, the constraints on factor loadings and intercepts are verified in a less stringent way, and the non-invariant factor loadings and intercepts are identified with respect to the group in which the non-invariance occurs. The concise comparison of the classical (exploratory) factor analysis, confirmatory factor analysis, multi-group factor analysis and multi-group factor analysis with alignment for a one-factor model with a special emphasis on the MI issue is presented in Table 5 in the Appendix. The process of the MI checking is illustrated in Fig. 1.

The MI properties of a scale are usually verified in a step-wise process starting from configural and ending with strong/strict MI, although the reverse procedure is also applicable. In each step, the fit of a specified MG-CFA model is assessed. From the broad range of goodness-of-fit statistics that are commonly employed in factor analytical models, we chose to report the root-mean-square error of approximation (RMSEA), the Tucker-Lewis index (TLI) and the comparative-fit index (CFI), as implemented in Mplus (Muthén and Muthén 2012). With respect to the RMSEA, values less than 0.08 indicate a satisfactorily low level of noise in the model (Browne and Cudeck 1992), and values less than 0.05 indicate a very low level of noise (Hu and Bentler 1999). Furthermore, the model is satisfactory if the CFI and TLI are greater than 0.95, although values greater than 0.90 only are also considered acceptable (Hu and Bentler 1999; Marsh et al. 2012).

However, based on previous studies (e.g. Hu and Bentler 1999; Kline 2011; Marsh et al. 2004, 2012), we treated these cut-off values only as rough guidelines. This is especially important when comparing a large number of groups/countries. In such a case, not only is little known about the performance of typically used fit measures (Rutkowski and Svetina 2013), but mechanical use of fit statistics can also easily lead to erroneous conclusions (Byrne and van de Vijver 2010).

## Data

As mentioned in the introduction, the aim of this study was to verify the country-specific MI properties of the GES based on the WVS data. With this respect, we replicate the approach used by Inglehart and Norris (2003), which determines the selection of the questions. The GES may be questioned because it comprises statements related to substantially different dimensions, such as labour market, family and institutions, which, as noticed by McDonald (2013), are differentially associated with outcomes, such as fertility. Nevertheless, this scale has still been used in numerous applications, and therefore, we treat it as a case study to highlight the problem of MI in the analysis of gender attitude concepts by measuring it by using a commonly used dataset.

The GES was originally constructed from the following five statements from the WVS (see Inglehart and Norris 2003); therefore, we also analysed data from the WVS (1994**–**1999) (World Values Survey Association 1995). The questions and answers are as follows:

- *V*61. *When jobs are scarce, men should have more right to a job than women. Answers*: 1*—agree*; 2—*neither agree nor disagree*; 3—*disagree*;
- *V*93. *Do you think that a woman has to have children in order to be fulfilled, or is this not necessary*? *Answers*: 1—*needs children*; 2—*it is not necessary*;
- *V*96. *If a woman wants to have a child as a single parent but she doesn*’*t want to have a stable relationship with a man, do you approve or disapprove*? *Answers*: 1—*disapprove*; 2—*it depends*; 3—*approve*;
- *V*101. *On the whole, men make better political leaders than women do. Answers*: 1—*strongly agree*; 2—*agree*; 3—*disagree*; 4—*strongly disagree*;
- *V*103. *A university education is more important for a boy than for a girl. Answers*: 1—*strongly agree*; 2—*agree*; 3—*disagree*; 4—*strongly disagree.*

Although our initial aim was to study gender norms in as broad a range of countries as possible, due to methodological issues described in the results section, in this study, we focused only on CEE countries. These are Albania, Bosnia, Bulgaria, Croatia, the Czech Republic, East Germany, Estonia, Hungary, Latvia, Lithuania, Macedonia, Poland, Romania, Serbia, Slovakia and Slovenia. Montenegro, because of a low sample size, was excluded from the analysis. Despite being based on this limited set of countries, the analysis is relevant as the knowledge about gender norms in the CEE countries is still low in comparison to Western Europe. Dramatic political and economic changes observed in this part of Europe in the 1980s and 1990s, as well as the considerable change in mainstream gender ideology, shaped the gender norms attitudes in a particular way (Fodor and Balogh 2010; Olson et al. 2007; Schmitt and Trappe 2010). In this paper, we show that although gender attitudes seem to be universal, the meaning that is attached to them differs not only between Western Europe and Central and Eastern Europe but also even among the CEE countries only.

## Modelling Strategy

To assess the MI, we used a multi-group factor analytical framework with and without alignment. It is worth noting that our goal was to first establish the configural invariance (see Asparouhov and Muthén 2014; Byrne and van de Vijver 2010). If configural invariance held, we proceeded to an analysis of the higher levels of measurement equivalence.

All of the CFA analyses were conducted using Mplus 7.11, and the descriptive statistics were performed in the IBM SPSS Statistics software (version 20). It must be noted, however, that the MG-CFA with alignment, as implemented in the Mplus, is a novel method, which brings about some limitations to our analysis. First, because for the time being, the MG-CFA with alignment operates for continuous and dichotomous variables only, we were forced to assume that our data are of a continuous nature. The alternative was to recode the data into dichotomous variables. Although the latter approach seems to be sounder, it would bring about a loss of comparability of the results obtained by other researchers, and the results obtained in this research between the approaches employed. Therefore, all calculations were performed using the robust maximum likelihood estimator (MLR, following Asparouhov and Muthén (2014)) which is one of the estimators in the analyses with continuous indicators in Mplus (Muthén and Muthén 2012). Second, the missing data imputations as well as weights were not taken into account because such options have not been implemented in the Mplus software with respect to MG-CFA with alignment.

We recognise that there is a disjuncture between our approach and the theoretically supported approach and that our choice is not considered best practice theoretically. However, this is intentional and reflects the best approach that is achievable in order to make comparisons between the approaches. Nevertheless, we are aware that it may influence the results and final conclusions.

## Results

In the first step, the unidimensionality of the five-item set of indicators was confirmed through an exploratory factor analysis of the pooled dataset (only one eigenvalue was higher than 1, i.e. 1.696). Additionally, the fit of this model was assessed by the confirmatory factor analysis. The model was found to fit the data very well (RMSEA = 0.032 < 0.08, CFI = 0.981 and TLI = 0.962 > 0.9). All of these results confirmed that the GES was one-dimensional and driven by a coherent set of indicators, despite describing considerably different aspects of gender equality as noticed in Sect. 4. This finding is in agreement with the results presented by other researchers (compare, for example, Inglehart and Norris 2003). However, the above conclusion only holds for the analysis conducted for the pooled dataset without applying a cross-country perspective. To verify whether the results are the same, when a cross-country perspective is taken into account, the MI properties of the GES with regard to the countries analysed were investigated.

As mentioned previously, we first analysed the configural invariance. This level of MI was not achievable with the data from the entire set of analysed countries from Central and Eastern Europe (RMSEA = 0.083 > 0.08, CFI = 0.864 and TLI = 0.771 < 0.9 in Table 1). Although we do not present the results here, we want to highlight that the configural invariance was not confirmed for the set of Western and CEE countries analysed together either. Therefore, it can be concluded that the construct being measured operates in a different way (e.g. is not one-dimensional) across the countries of interest, and this reasoning applies to both (1) the set comprising the Western European and CEE countries and (2) the CEE countries only.

**Table 1:** Steps in the process of establishing the measurement invariance in multi-country analysis—fit statistics for a one-factor solution in multi-group confirmatory factor analysis

Countries	RMSEA	CFI	TLI
Pooled dataset (confirmatory factor analysis)			
All 16 countries	0.032	0.981	0.962
Multi-country analysis (multi-group confirmatory factor analysis)			
Configural invariance			
All 16 countries	0.083	0.864	0.771
CEE countries belonging to the EU and Albania	0.057	0.933	0.901
Full weak measurement invariance			
CEE countries belonging to the EU and Albania	0.175	0.000	0.000

*Note* *RMSEA* the root-mean-square error of approximation; *CFI* the comparative-fit index; *TLI* the Tucker-Lewis index; *RMSEA* < 0.08 indicates a satisfactorily low level of noise in the model; *CFI* > 0.95 and *TLI* > 0.95 indicate satisfactorily level of model fit; *CFI* > 0.90 and *TLI* > 0.90 indicate acceptable level of model fit; *CEE* Central and Eastern European countries, *EU* European Union

The question is whether misfit of the configural model is due to the cultural influence of particular countries, to the non-equivalence of particular items across countries or to a combination of both. The analysis of the country chi-square contribution to the fit statistics revealed that the CEE countries that stood out considerably were East Germany, Latvia and four former Yugoslav republics, i.e. Slovenia, Serbia, Macedonia and Bosnia.^1^ Further analysis revealed also that the CEE countries belonging to the European Union (excluding Latvia and Slovenia) and Albania were shown to be configurally invariant (RMSEA = 0.057 < 0.08, CFI = 0.933 and TLI = 0.901 > 0.9 in Table 1). It implies that the notion of gender equality may be operationalised in the form of one-dimensional GES within these countries. However, the weak MI was not established for the group of countries with configural invariance (RMSEA = 0.175 > 0.08, CFI = 0.000 and TLI = 0.000 < 0.9 in Table 1), which implies that this group of countries does exhibit neither full strong MI nor full strict MI.

Because the analysis was conducted on a large set of countries (groups), to get a more realistic picture, we investigated the issue of weak and strong MI with the MG-CFA with alignment. The computations were performed on the groups of countries exhibiting a satisfying level of configural invariance, which, as suggested by Asparouhov and Muthén (2014), is a prerequisite to obtaining reliable results.

Our results (Table 2) show three countries for which non-invariant factor loadings were recorded. It was the factor loading related to the question V93 for Poland, Albania and Croatia and the factor loading corresponding to the question V96 for Croatia. The incidences of non-invariant intercepts were more numerous. With regard to V61 and V101, there were two countries for which the non-invariance occurred, i.e. Hungary and Poland with respect to the former and Albania and Croatia with respect to the latter. Regarding the remaining questions, the number of countries with non-invariant intercept were six, six and four for V93, V103 and V96, respectively.

**Table 2:** Countries with non-invariant loadings and intercepts based on the multi-group confirmatory factor analysis with alignment

Question	Country in which non-invariant loading occurs	Country in which non-invariant intercept occurs
V61	None	Hungary, Poland
V93	Poland, Albania, Croatia	Hungary, Poland, Bulgaria, Romania, Albania, Croatia
V96	Croatia	Hungary, Poland, Bulgaria, Romania
V101	None	Albania, Croatia
V103	None	Hungary, Czech Republic, Bulgaria, Albania, Croatia

These results show that the least problematic questions were V61 and V101, which both relate to the comparison of the situation of men and women. The most problematic questions were V96 and V93 (both having non-invariant factor loadings and intercepts), which are both about attitudes towards having children. The most problematic countries were, in turn, Croatia for which the violation of invariance occurred five times (two factor loadings and three intercepts), and Poland, Albania and Hungary with violations of invariance occurring four times. In the case of Poland and Albania, it happened once for factor loading and three times for intercept, in the case of Hungary—four times for intercepts.

All of the results presented above imply that although the GES has the same meaning within a certain group of countries (i.e. the selected CEE countries belonging to the European Union and Albania), which is guaranteed by the good fit of the GES model with configural MI, its scaling properties with respect to the measurement unit and the reference point are not perfectly the same. Thus, it is not advisable to use the GES scores obtained using either classical (exploratory) factor analysis (i.e. factors scores), principal component analysis or by summation of the codes of answering categories to 1) make valid comparisons of the relationships between the GES and other variables because the actual relationships between gender equality and other variables might be different from those reflected by the correlation or regression coefficients; 2) make comparisons of the gender attitudes between the countries using the GES or to determine country rankings with respect to this scale because the real ordering may be completely different.

We try to visualise the issue below. Correction for non-invariance of certain factor loadings and/or intercepts is necessary. It can be obtained, for example, from the MG-CFA with alignment. Such corrected scale means as well as factor scores (i.e. quantifications of the GES scale for each individual) are available in Mplus and are presented in Tables 3 and 4.

**Table 3:** Comparison of the results obtained with the classical (exploratory) factor analysis and the multi-group confirmatory factor analysis with alignment

Country	Factor mean		Correlation with age (Pearson correlation coefficient)	
	Classical (exploratory) factor analysis	Multi-group confirmatory factor analysis with alignment	Classical (exploratory) factor analysis	Multi-group confirmatory factor analysis with alignment
Czech Republic	−0.047 (5)	0.336 (1)	−0.186** (4)	−0.204** (3)
Hungary	−0.013 (3)	0.319 (2)	0.010 (8)	0.090 (10)
Lithuania	−0.015 (4)	0.296 (3)	−0.203* (3)	−0.181** (5)
Croatia	0.201 (1)	0.249 (4)	0.050 (10)	0.023 (8)
Estonia	−0.155 (7)	0.186 (5)	−0.156** (7)	−0.156** (7)
Bulgaria	0.004 (2)	0.170 (6)	−0.239** (2)	−0.206** (2)
Poland	−0.163 (8)	0.054 (7)	−0.337** (1)	−0.311** (1)
Slovakia	−0.247 (9)	0.010 (8)	−0.163** (6)	−0.174** (6)
Albania	−0.097 (6)	0.000 (9)	0.028 (9)	0.026 (9)
Romania	−0.334 (10)	−0.151 (10)	−0.176** (5)	−0.185** (4)

*Note* rank in brackets; ** correlation is significant at the 0.01 level (two-tailed); * correlation is significant at the 0.05 level (two-tailed)

**Table 4:** Factor mean comparison

Country	GES mean-multi-group confirmatory factor analysis with alignment	Country with significantly smaller GES mean
Czech Republic	0.336	Bulgaria, Poland, Slovakia, Albania, Romania
Hungary	0.319	Poland, Slovakia, Albania, Romania
Lithuania	0.296	Poland, Slovakia, Albania, Romania
Croatia	0.249	Poland, Slovakia, Albania, Romania
Estonia	0.186	Albania, Romania
Bulgaria	0.170	Slovakia, Albania, Romania
Poland	0.054	Romania
Slovakia	0.010	None
Albania	0.000	None
Romania	−0.151	None

*Note* Significance level 0.05

To better visualise both the issue and the consequences of ignoring non-invariance problem in this section, we compare the results obtained using the classical (exploratory) factor analysis and MG-CFA with alignment. We compare 1) the mean factor values; 2) relationships between the GES scale and the age of respondent;

First, we focus on the comparisons of the mean factor values. In Table 3 (left panel), we present the mean values of the GES scores calculated for each country. The mean values based on the classical (exploratory) factor analysis scores are calculated as an average of the GES scores obtained by persons from a given country. The mean values based on the MG-CFA with alignment are directly estimated from the model. As seen in Table 3, the values obtained through each method differ significantly. Regarding the scores, it was expected because each model was estimated separately and with different specifications. However, with respect to the rankings, we see that although the ranks obtained using two methods are correlated (Spearman rank correlation *r*_S_ = 0.648), the ordering is considerably different. The median shift between ranks amounts to 1.5 and the average shift equals 2. The only country that ranks exactly the same is Romania—the last one in the ranking. On the other hand, the best scoring Czech Republic is only fifth when classical (exploratory) factor analysis is employed.

Second, we draw attention to differences in relationships between the GES scale and other variables. As an example, we take the respondent’s age, and we check the correlation between the GES scores and his or her age. This is done for each country (Table 3, right panel), and it appears that the differences in the strength of the relationship are similar with respect to the level, direction and significance level. In both approaches, only the negative relationships are statistically significant. The strongest and most negative relationship between age and gender equality is recorded in Poland, Bulgaria (both medium scoring with respect to the average level of the GES) and the Czech Republic (leader with respect to the average level of the GES). The weakest relationship (though positive) is recorded in Croatia (medium scoring with respect to the average level of the GES), Albania (second worst) and Hungary (second best).

The above findings are not surprising when the results of MG-CFA with alignment are taken into consideration. We recall that for only two out of five analysed questions non-invariant loadings were spotted (see Table 2), whereas non-invariant intercepts were recorded for all analysed questions. Then, the total number of non-invariance occurrence was higher in the case of intercepts (19) than with respect to loadings (4). Relatively low number of the non-invariant factor loadings,^2^ which are necessary to compare across groups in a valid way the relationships between factor scores/scale scores and other observable variables, explains why the differences in the correlation coefficient (see Table 3, right panel) are so small. In other words, not correcting for non-invariant factor loadings has a small impact on the correlations because the number of required corrections is low.

This is not the case regarding the comparisons of means (see Table 3, left panel). We recall that in order to correctly compare means, both factor loadings and intercepts should be invariant across groups. In the case of the GES, although the number of non-invariant loadings is low, the number of non-invariant intercepts is considerable. Therefore, not correcting for the lack of non-invariance influences more the outcomes and results in considerably diverse rankings.

Another useful feature of the MG-CFA with alignment implemented in Mplus is the possibility of calculating the statistical significance of the difference between the latent means. In Table 4, we present the comparison of the average levels of the GES. The highest average level of the GES is spotted in the Czech Republic, and in this respect, it is significantly higher than in the five lowest scoring countries, namely Bulgaria, Poland, Slovakia, Albania and Romania. The four lowest scoring countries with respect to the GES, i.e. Poland, Slovakia, Albania and Romania, lay significantly behind the four best scoring countries, namely the Czech Republic, Hungary, Lithuania and Croatia.

These results are not in line with the results obtained by Inglehart and Norris (2003). Analysing only a common set of countries, namely those identified in this study as possessing the MI properties with respect to the GES, we see that the rankings differ. In the approach taken by Inglehart and Norris, the leading position among the selected CEE countries belongs definitely to Croatia, which is followed by Lithuania. Then, the group of countries scoring at a similar level, despite being considerably lower, can be spotted. These are the Czech Republic, Bulgaria, Hungary, Albania and Estonia. The lowest ranks belong to Romania, Slovakia and Poland. In our approach, the Czech Republic definitely leads. Croatia is fourth, although its score is not significantly lower than the score of the Czech Republic (see Table 4). Regarding the countries lagging, in our approach, this group comprises not only Romania, Slovakia and Poland—identified as the lowest scoring by Inglehart and Norris—but also Albania, which in the approach of Inglehart and Norris scores very similarly to the Czech Republic and Hungary. However, in our approach, the score obtained by Albania is significantly lower than the scores of these two countries.

We claim that the differences described above are very likely to result from not accounting for the lack of MI of particular factor loadings and intercepts. We are aware, however, that they might also have been brought about by differences in the calculations. Among such differences, we include weighting or imputations, which were not taken into account by us or other differences that we are not aware of. However, regarding the weights and imputations, in order to verify the robustness of our results, we also performed exploratory factor analysis and MG-CFA with the weights (we recall that using weights in the MG-CFA with alignment is not feasible in Mplus). We must state that the results, despite being numerically different, were similar from a substantial point of view.

## Conclusions and Discussion

In this paper, we answer to the call of Davis and Greenstein (2009) for research on the validity and reliability of the measures used to capture gender ideology. Although their call was linked more to longitudinal and life course-related studies, we argue that it is also valid for research oriented towards cross-country comparisons. In this study, we concentrate on MI, which is an indispensable characteristic for reliable and valid comparisons of a latent phenomenon.

In our opinion, assessment of the MI of gender-related survey data has not received sufficient attention. Therefore, we attempt to show that ensured MI properties of the composite index measuring gender role attitudes in the cross-country studies are indispensable in regard to providing reliable results. Although the assessment conducted in this study was performed with respect to countries, we want to stress that measurement equivalence might and should be examined based on other classification variables used in a study. For example, we are convinced that the MI of gender role attitudes, which significantly differ between males and females and evolve among generations, should be analysed with respect to gender and cohort.

Tests of cross-country equivalence were performed using a factor analytical framework and through tests of (1) the full MI of the scales and (2) the MI with alignment. The results presented a complex picture, which indicated that the gender equality concept is not fully comparable across all of the surveyed countries and that the use of the data for secondary data analysis between all of the countries is usually not perfectly feasible. To be more specific, we clearly showed that the composite indicator of gender equality, i.e. the GES but also others, calculated as a sum of the answers or factor scores obtained through classical factor analysis or principal component analysis used (1) to compare gender attitudes between countries or (2) to present country classifications, should always be verified with respect to its measurement equivalence. In the case of its absence, the conclusions drawn may be biased. Then, the composite indicator of gender equality, i.e. the GES, expressed as factor scores obtained through classical factor analysis and used in a regression framework may provide less biased results than expected. This finding, however, only applies to the correlation between the GES and the age of an individual. Therefore, it does not influence our strong belief that ensuring the measurement properties of the composite scale is compulsory.

Our results show that although the gender role attitudes seem to be universal, the meaning that is attached to them differs not only between Western Europe and Central and Eastern Europe but even within the group of CEE countries. This conclusion is supported by the lack of the configural MI among all analysed countries. We then discovered that only the CEE countries currently belonging to the European Union and Albania exhibit a configural MI, which implies that they share a common understanding of the concept of gender attitudes. This subset, however, generally fails to show full weak MI and definitely fails to show full strong and full strict MI. However, under the “not perfect” MI framework obtained through the MG-CFA with alignment, we succeeded in showing that for selected CEE countries, comparisons of the country rankings with respect to the GES are valid. However, this was the case only after a correction for non-invariance of certain factor loadings and/or intercepts.

The implications of the above findings are that only in CEE countries such as Albania, Bulgaria, Croatia, the Czech Republic, Estonia, Hungary, Lithuania, Poland, Romania and Slovakia, the GES measures a homogenous phenomenon. In other countries, this scale does not measure either the same construct or not exactly in the same way across countries. However, this implication does not preclude the existence of the MI properties of the GES among another group of countries, such as Western European or the former Yugoslav republics. It must be noted that in such cases, GES scores would not be comparable between the groups of countries analysed because they would measure either different, despite probably somehow related, phenomena, or, even if they measure the same phenomena, the measurement processes in these groups of countries will not be the same. From a technical point of view, this might be due to conceptual misspecifications resulting from the fact that the WVS data represent multi-cultural rather than monocultural populations. Therefore, cumulated small and inconsequential differences in the parameters, or combination of both, might be expected (Byrne and van de Vijver 2010).

From a substantial point of view, taking into consideration the opinion that gender roles are deeply connected with a society’s social organisation (Sackmann 1998), the findings show that the gender attitudes in CEE societies and in Western European societies may be shaped by qualitatively different processes. This, in turn, supports the suggestion made by Pfau-Effinger (1998) and Raabe (1998) that although some phenomena seem to be universal, the meaning attached to them, often resulting from well-embedded and long lasting beliefs related to institutions and social structures, may be different. We argue, therefore, that as CEE countries for quite a long period were characterised by a particular configuration of cultural tradition, social structure and social institutions, their societies underwent a different track in the formulation of gender attitudes then the Western European societies did. For example, Western European traditional division of labour based on a male breadwinner and female homeworker before the1970s was followed by the women’s liberation movement resulting in more egalitarian gendered division of labour both on the labour market and at home. On the other hand, in the socialist countries in the 1960s–1980s, the state-promotion of the dual-earner model with the state childcare (Pfau-Effinger 1998) was common. Then, with the fall of the Iron Curtain and drastic social reforms, women’s situation changed both at home and on the labour market. They became more dependent on male earners and more focused on the family. These considerably different dualistic processes, in turn, might have influenced the meaning that is attributed to the gender role attitude concept, which, following Jahnert et al. (2001), in the CEE may bear certain special features. This conclusion is consistent with the conclusion made by Fortin (2005) that not only are some attitudes with respect to the traditional gender roles formed in youth and strongly related to religious ideology but also that they may not be sensitive to experiences from the adult life. It is also supported by findings provided by Steinhilber (2003) who showed that in CEE countries in the early 2000s, egalitarian gender attitudes seem to be considerably common when analysed at face value, but in practice, it often meant preferential treatment for women to compensate for their disadvantageous status in the labour market.

Additionally, the technical issue related to the influence of even subtle differences in translation on the observed differences in the meaning should not be neglected. The same applies to technical difficulties related to the answers for the question *V*61. *When jobs are scarce, men should have more right to a job than women*, which, if positive, may indicate the belief that the man is the main breadwinner and earns higher wages without implying any discriminatory attitude.

This study makes the following contributions to the field of gender role attitudes. First, as mentioned above, our study shows that we can compare countries within the group of CEE countries. The highest levels of the gender equality attitude measured by the GES are observed in the Czech Republic, Hungary, Lithuania and Croatia. It is significantly higher than the gender equality attitudes recorded in the lowest scoring Poland, Slovakia, Albania and Romania. This means that Czech, Hungarian, Lithuanian and Croatian societies are considerably more egalitarian with respect to gender equality than Polish, Slovakian, Albanian and Romanian societies. Although this ranking differs from the ones provided by other researchers without taking into account MI issues, it enabled us to confirm the positive relationship between gender equality attitudes and economic development (the results are not presented), which was reported by Olson et al. (2007). It means that more egalitarian views are associated with favourable economic conditions, i.e. higher Gross Domestic Product (GDP) per capita, lower unemployment rate and lower inflation. We also found a positive relationship between the gender role attitude and GDP growth. In the study by Olson et al. (2007), it was reported as negative, although insignificant. Both findings may be correct because due to convergence, poorer countries (in terms of GDP) tend to grow at faster rates than richer countries. However, poorer countries are also shown to have societies with more traditional views on gender roles. Taking into consideration that the set of analysed countries here is quite homogenous in terms of GDP and in the analysis by Olson et al. (2007), the US was analysed next to the set of CEE countries, and both findings are probable. We must state, however, that due to the small sample size, these findings should be treated with caution and require confirmation in future studies.

Second, taking into consideration all reservations related to the limited comparability of results obtained with different sets of questions from different surveys and other sets of countries under investigation, we attempted to perform such comparisons. Comparing the results of Philipov (2008) and Luck and Hofacker (2003) with our results, we found support that East Germany, despite being frequently classified as a CEE country, stands out from other countries in this group considerably. We also spotted substantial differences with respect to the position of Hungary. Hungarian people were shown by Philipov and by Luck and Hofacker to have strongly traditional gender role attitudes, whereas in our study, they have the second-most egalitarian gender attitude, following the Czech. Regarding the Czech people, our results are similar to the results of Luck and Hofacker. In both these studies, the Czech belong to the most egalitarian CEE societies. However, according to Luck and Hofacker, the Polish and Slovak belong to this group as well. Our findings are different, namely that Poland and Slovakia are among the countries with significantly more traditional gender attitudes. This is in contrast to Bulgaria, which in our study is moderately egalitarian but very traditional in the study by Luck and Hofacker. On the other hand, we confirmed the relative standing of Romania. Similar to the findings of Philipov, we found that Romania is very traditional with respect to gender role attitudes. Third, our results may shed light on the contradictory results on the relationships between men’s gender attitudes and fertility obtained by among others Puur et al. (2008) and Westoff and Higgins (2009). Their findings were based on the correlation-like analyses built on the assumption that at least full weak MI holds. This lack of weak MI, if confirmed for the gender role summary index, time points, set of countries and men—corresponding to the specifications of the analyses performed by the authors—may have been the source of disjunctive findings.

To conclude, we note that our paper aims only to detect, not to determine, the cause of non-invariance. As such, we advocate an approach whereby once measurement non-invariance (under full, classical partial or with alignment framework) is detected, in-depth analyses are necessary to locate its source. Of several possibilities, we first opt for consultation with cultural studies experts or linguists to examine potential sources of variability (Rutkowski and Svetina 2013). We join Fortin (2005) in her call for further research on the processes involved in evaluating gender attitudes. We suggest examining through multilevel structural equation modelling, namely two-level confirmatory factor analysis, the reasons for measurement non-invariance. To this end, we propose investigating whether contextual country-level covariates could explain why MI was not achieved [for the methodological details of the approach see Hox (2010) and for the application—Davidov et al. (2012)]. In such an approach, country-level information is treated as a possible source of bias (non-invariance) and is used (instead of personal level information) to explain the differences in questions whose performance displays large differences across countries.

## Appendix

See Tables 5 and 6.

**Table 5:** Comparison of the classical (exploratory) factor analysis, confirmatory factor analysis, multi-group confirmatory factor analysis and multi-group confirmatory factor analysis with alignment

	Classical (exploratory) factor analysis/confirmatory factor analysis^a^	Multi-group confirmatory factor analysis (MG-CFA)	Multi-group confirmatory factor analysis with alignment
Application	Only one population/group is analysed; To test whether measures of a construct are consistent with a researcher’s understanding of the nature of that construct; in other words, to test whether the data fit a hypothesized measurement model that is based on a theory and/or previous research	A few populations/groups are analysed; To test whether measures of a construct are consistent with a researcher’s understanding; To measure the construct in different populations	Multiple populations are analysed (<100); To test whether measures of a construct are consistent with a researcher’s understanding; To measure the construct in different populations
Model specification	$X_{ij} = \tau_{j} + \lambda_{j} \eta_{i} + \varepsilon_{ij}$ (1), where $X_{ij}$-observed scored X of variable *j* for person *i*; $\tau_{j}$-intercept of variable *j*; $\eta_{i}$-latent factor score of person *i*; $\lambda_{j}$-factor loading corresponding to variable *j*; $\varepsilon_{ij}$-unique factor score or residual	$X_{ijg} = \tau_{jg} + \lambda_{jg} \eta_{ig} + \varepsilon_{ijg}$ (2), where $X_{ijg}$-observed scored X of variable *j* for person *i* in group *g*; $\tau_{j}$- intercept of variable *j*; $\eta_{i}$-latent factor score of person *i*; $\lambda_{j}$- factor loading corresponding to variable *j*; $\varepsilon_{ij}$-unique factor score or residual	The same as Eq. (2) The same as for the MG-CFA
Interpretation	For each statement *j*, the Eq. (1) is to predict the item score *X* for an individual *i* as a linear combination of the statement-specific intercept $\tau_{j}$, the factor score $\eta_{i}$ times the statement-specific factor loading $\lambda_{j}$ and a random error component $\varepsilon_{ij}$; The statement-specific intercept $\tau_{j}$ relates to the score of the statement *j* when the factor score of the latent variable $\eta$ equals 0. The statement-specific factor loading $\lambda_{j}$ indicates the change in the score of the statement j when the score of the latent variable $\eta$ of an individual *i* is changed by one unit	For each statement *j,* the Eq. (2) predicts the item score *X* for an individual *i* from the group *g* as a linear combination of the statement-specific intercept $\tau_{jg}$, the factor score $\eta_{ig}$ times the factor loading $\lambda_{jg}$ and a random error component $\varepsilon_{ijg}$; The subscript *g* in the formula indicates that the parameters $\tau$ and $\lambda$ are allowed to vary across groups, i.e. depend on the group membership of the individual; In the MG-CFA the mean and variance of the latent variable $\eta$ are estimated and differ depending on the group membership	The same as for the MG-CFA
Measurement invariance issue	Because score of each statement is a function of only a latent factor $\eta$ and not of a group membership, it is silently assumed that the underlying construct is comparable across groups and it can be measured comparably across groups, i.e. it is characterised by full configural, weak and strong measurement invariance	Because score of each statement is a function of both a latent factor $\eta$ and a group membership in order to compare the construct across groups the measurement invariance feature of the model is needed; In order to ensure 1) configural measurement invariance, it is necessary to obtain well-fitted MG-CFA model with unconstrained factor loadings and intercepts; unfortunately in such a model factor, means and variances are not identified and are typically set to 0 and 1, respectively 2) weak measurement invariance, it is necessary to verify the condition $\lambda_{1} = \lambda_{2} = \ldots = \lambda_{g}$ 3) strong measurement invariance, it is necessary to verify two conditions $\lambda_{1} = \lambda_{2} = \ldots = \lambda_{g}$ and $\tau_{1} = \tau_{2} = \ldots = \tau_{g}$	The alignment process aims to minimize the amount of non-invariance by 1) estimation of the configural model (i.e. model with the same number of factors and same pattern of zero factor loadings in all groups) in which loadings and intercepts are free across the groups, factor means are fixed at 0 and factor variances are fixed at 1; 2) alignment optimisation where the estimation of the model is conducted under the assumption that the number of measurement non-invariance parameters is as small as possible; 3) adjustment of the factor means and variances in line with the optimal alignment. The relationship between models in points 1 and 2 is similar to the relationship we observe in factor analysis between unrotated and rotated (which simplifies the loading matrix) solutions; specifically, the alignment aims at finding a solution with a few large non-invariant parameters and many invariant parameters rather than many medium-sized non-invariant parameters. In Mplus, two alignment optimisations are available: *fixed* and *free.* The fixed option assumes that the mean of the latent factor in the first analysed group is equal to 0. The free option, instead, assumes that all means of the latent factor are estimated freely. Whenever more than two groups are compared, *free* alignment provides more accurate estimates. However, in practical, applications often appear that *free* alignment may be poorly identified (warning obtained automatically in Mplus) and the application of the *fixed* alignment (with the mean of the latent factor in the group where it is the smallest fixed as 0) is suggested. Estimated model has the same fit as the configural model
Fit measures	CFI, TLI, RMSEA	CFI, TLI, RMSEA	CFI, TLI, RMSEA to assess the configural model
Software	Classical (exploratory) factor analysis: SPSS, Statistica, SAS, Mplus (the only one where the fit statistics are available) Confirmatory factor analysis: LISREL, EQS, SPSS-Amos, Statistica, SAS, Stata, Mplus	LISREL, EQS, SPSS-Amos, Stata, Mplus	Mplus

*Note* Based on the information presented by Muthen and Asparouhov (2013); Asparouhov and Muthén (2014) and Segeritz and Pant (2013)

^a ^Classical (exploratory) factor analysis and confirmatory factor analysis are perfect counterparts only for a one-factor model

**Table 6:** List of countries analysed with regard to the Gender Quality Scale and amount of missing values

Country	Sample size	V61 (%)	V93 (%)	V96 (%)	V101 (%)	V103 (%)
Albania (CEE)	999	6.21	2.10	9.31	7.51	4.60
Bulgaria (CEE)	1,072	4.01	8.96	3.82	15.76	12.87
Croatia (CEE)	1,196	0.59	7.69	1.76	4.60	1.92
Czech Republic (CEE)	1,147	1.13	7.06	3.57	7.76	4.10
Estonia (CEE)	1,021	1.18	5.39	1.08	4.41	3.43
Hungary (CEE)	650	0.77	6.31	1.23	5.85	2.15
Lithuania (CEE)	1,009	4.06	11.4	4.06	10.11	9.32
Poland (CEE)	1,153	3.56	6.24	3.82	16.13	14.74
Romania (CEE)	1,239	3.47	6.21	4.84	11.54	12.27
Slovakia (CEE)	1,095	1.10	4.38	3.47	6.94	5.39

*Note* Average sample size 1058

## Footnotes

1 To establish a set of countries characterised by the configural MI with respect to the GES, using exploratory factor analysis for each country separately, we checked the fit of (1) one-factor model, then, (2) in the case of low fit, two-factor models. In general, the results corresponded to these based on the analysis of the Chi square contribution of each country to the model fit supporting their robustness.

2 We recall that equal factor loadings across the groups (obligatory condition for weak MI) are necessary.
